# Family Trajectories and Well-being of Children Born to Lone Mothers in the UK

**DOI:** 10.1007/s10680-017-9420-x

**Published:** 2017-03-23

**Authors:** Elena Mariani, Berkay Özcan, Alice Goisis

**Affiliations:** 1 Department of Social Policy, London School of Economics, Houghton Street, WC2A 2AE London, UK; 2 Max Planck Institute for Demographic Research, Rostock, Germany

**Keywords:** Lone mothers, Child well-being, Family trajectories, UK

## Abstract

We investigate how lone mothers’ heterogeneity in partnership trajectories is associated with children’s well-being. We use data from the Millennium Cohort Study, which follows a large sample of children born in the UK in 2000–2002. We divide children who were born to lone mothers into four groups based on their mothers’ partnership trajectories between birth and age seven, which cover more than 80% of these children’s family experiences. We then analyse how these trajectories are associated with markers of health, cognitive and socio-emotional outcomes measured at around age seven. We find that compared to the children that live continuously with lone mothers, children whose biological father stably joined the household have better cognitive and socio-emotional outcomes. In contrast, children in trajectories characterised by living with a stepfather or who experienced biological father joining in the family followed by biological parents’ dissolution had outcomes similar to children living continuously with lone mothers. The results underscore the importance of treating children born to lone mothers as a heterogeneous category.

## Introduction

There has been a large amount of research on the relationship between family structure and child development (see Amato 2000, 2001, 2010; Sigle-Rushton and McLanahan 2004; McLanahan et al. 2013 and Bernardi et al. 2014 for recent reviews of the literature). Most of these studies argued that children who grow up in a household with two married biological parents do better overall than those growing up with a single mother (e.g. Amato 2001) and that parental separation is negatively associated with a variety of child outcomes (e.g. Amato 2010). Overall, the evidence suggests that there is a negative association between the father’s absence and child well-being (see McLanahan et al. 2013 for a recent review).

The existing literature has compared the well-being of children growing up with a lone mother with children in stable biological families. However, the diverse family trajectories of children born to a lone mother (i.e. who was neither married nor cohabiting when the child was born) and how these trajectories are related to their well-being are largely neglected. The contribution of this study is to analyse how heterogeneity of family life experiences among lone mothers is associated with their children’s well-being and development.

We use data from the Millennium Cohort Study (MCS), a longitudinal cohort study of children born in the UK between 2000 and 2001. We compare a set of outcomes measured at age seven of a sample of children born to lone mothers but who subsequently experienced different family trajectories. The family trajectories we consider are differentiated by whether the lone mother remained single or eventually formed a union with the biological father of the child or with another partner, as well as by the stability associated with each of these relationship states up to the time of the interview (i.e. age seven). These trajectories collectively describe around 82% of all of the family trajectories experienced by children born to a lone mother in the UK up to age seven.

We analyse three key domains of child well-being: health (obesity), cognitive development (test scores on word recognition, number skills and pattern construction) and socio-emotional well-being to describe, for the first time outside the USA, the association between the family trajectories experienced by children born to lone mothers and their well-being (see Heiland and Liu 2006; Cavanagh and Huston 2006 and Craigie et al. 2012 for similar studies that use data from the USA). We focus on outcomes measured at age seven, since family transitions and instability early in life may be particularly detrimental for children’s development at later ages (Cavanagh and Huston 2008) and because evidence suggests that these markers of child well-being are predictors of well-being later in life. For example, cognitive skills are found to be strong predictors of future earnings, labour marker attachment and other social behaviours (e.g. Heckman et al. 2006). Carneiro et al. (2007) have showed that markers of socio-emotional well-being in childhood are predictors of a wide range of outcomes, including the likelihood of obtaining a degree, wages, smoking behaviour, teenage pregnancy, and involvement with crime. Finally, Reilly and Kelly (2011) have found that obesity during childhood is associated with an increased risk of premature mortality and of developing cardiometabolic morbidity (e.g. diabetes, heart diseases and stroke).

Two additional observations of the literature motivate our study. First, most of what we know about children born to lone mothers and the association between their subsequent family transitions and well-being comes from a handful of studies which rely on data sets from the USA. These data have specific features that make them representative of only a certain period^1^ (Stewart 2006) or of certain population subgroups^2^ (Sweeney 2010), complicating the issue of the transferability of findings to children currently living in other country contexts (Heiland and Liu 2006; Cavanagh and Huston 2006; Craigie et al. 2012 which use the US data.). Few empirical studies focus on children born to lone mothers using representative data sets in Europe (e.g. Turunen 2011 using Swedish data and Kiernan 2006; Flouri and Malmberg 2012; Kiernan et al. 2011 using British data sets), and none explicitly focuses on the question of how various family trajectories of children are related to their well-being. This is unfortunate because the number of children born to lone mothers has been growing substantially in Europe, and in the UK in particular (Andersson 2002; Kiernan 2006). In 2014, according to the Office of National Statistics, approximately 16% of all children in the UK were born to a lone mother (ONS 2014), which is close to what is documented in the USA (Andersson 2002). These features make the UK a particularly fruitful context for our analyses.

Second, from a theoretical point of view it is not clear, a priori, whether and how diverse family trajectories are associated with child outcomes. Previous studies in the USA have shown that there is considerable variation in the subsequent union formation patterns and relationship stability levels of lone mothers (e.g. Bzostek et al. 2007). This variation is associated with differences in the mothers’ income and wealth accumulation levels (e.g. Painter et al. 2015), later health outcomes and psychological well-being (e.g. Lichter et al. 2003; Williams et al. 2011). Variation in the relationship trajectories of lone mothers may not only affect themselves but may also be associated with their children’s well-being.

In the next section, we summarise the main theoretical arguments about why different family trajectories may be associated with differences in child outcomes. We focus on *four* most commonly experienced family trajectories of children born to lone mothers: first, children may live continuously with a lone mother until age 7, which constitute our reference trajectory. Second, they may experience their biological father moving in, forming a stable union with their mother. Third, the biological father may leave after moving in with their mother, and thus, these children may experience both parental union formation and subsequent dissolution before age 7. Fourth, their mother may form a stable union with someone else. In Sect. 3, we describe in detail the characteristics of these family trajectories and the strategy we use, along with our data and analytical sample. In Sect. 4, we elaborate on the applied measures for each of the three domains of well-being (health, socio-emotional and cognitive). It is likely that children born to lone mothers with different family trajectories differ in background characteristics, which explain potential differences in their outcomes. In Sect. 5, we explore the association between the three categories of child outcomes and the family trajectories experienced by children born to lone mothers before and after the adjustment for background characteristics. To provide context and discussion for our results, we also compare children of lone mothers in different trajectories to children growing up in stable biological families.

## Theoretical Background and Previous Literature

There may be a range of mechanisms that underlie the association between family trajectories and the outcomes of children born to lone mothers which may also operate differently across child outcomes (Thomson and Mclanahan 2012). Three theoretical arguments have been commonly used to understand the role of family structure on child outcomes: *changes in family resources*, *instability*-*stress* and *social control*. Among these, the family resources perspective and instability-stress theory have played a more prominent role in explaining how family structure and its changes are related to socio-emotional behaviour and cognitive skills of children (Mitchell et al. 2015). *Social control* and *social learning* theories have been considered useful for understanding the physical health outcomes of children (e.g. Bzostek and Beck 2011; Reczek et al. 2014). The latter theories have also been used for studying problem behaviours during teenage years rather than early childhood (e.g. Wadsworth 2000).

Theories of *family resources* hark back to Coleman’s (1988) point that parents accumulate and invest in financial, human and social resources. These resources are found to be crucially important for the educational outcomes and for the socio-emotional behaviour of children (e.g. Thomson et al. 1994). This perspective treats resources broadly to include monetary, social capital and parental time resources available for children and predicts that these resources reduce following a union dissolution and are lower in lone parent households (Thomson et al. 1994; Amato 2001; Sigle-Rushton and Mclanahan 2004). Fathers’ exits often imply dissolution of economies of scale in the household, and hence an increase in economic costs, and a decline in family income (Becker 1981). They also lead to the loss of parental trust and connections available for children (Mitchell et al. 2015). There is large evidence supporting these arguments showing that mothers and children who experience a marital break-up are more likely to fall into poverty than those who grow up in intact families (e.g. Mclanahan 1985; Holden and Smock 1991 and see the studies in Amato 2001 and Kiernan and Mensah 2011 for the UK).

*Family instability-stress* theories predict that family changes in itself are also harmful for children (e.g. Osborne and McLanahan 2007; Sweeney 2010). These theories, which built on the social stress theory of family stability (e.g. George 1993), argue that changes in family structure and resources may create additional stress for both parents and require children to adjust to the new environment (Cavanagh et al. 2006; Wu and Martinson 1993). Thus, instability and change generated by family transitions may have independent negative effects on child outcomes that go beyond the effects of the household structure at each point in time (Thomson and McLanahan 2012). Overall, these theories suggest a negative effect of a father’s exit on children’s socio-emotional behaviour and cognitive skills, which is supported by empirical evidence (McLanahan et al. 2013). *Family resources* and *family instability-* *stress* theories consistently predict worse outcomes for children in case of a father’s exit. However, these theories predict ambiguous overall effects for family trajectories when they include new union formations and fathers’ entries on children’s outcomes (Mitchell et al. 2015) as we outline below.

### Does Entry of the Father in the Household Matter?

The entry of the father or a father figure in the household may lead to an increase in the household’s income and economic resources and to a reduction in the family’s time constraints and monetary costs due to improved economies of scale (e.g. Thomson et al. 1994; Mclanahan and Sandefur 1994). Thus, according to the *family resource* theory, the trajectories in which the mother forms a union (with the biological father or with a new partner) should, all other things being equal, have a positive effect on children’s outcomes. Furthermore, the addition of a second parental figure may provide childrearing support, as well as emotional support to both the lone mother and the children (Booth and Amato 1994).

However, according to *family instability-stress* theory, these benefits might be, at least partially, offset by further instability in the family, especially if the child has difficulties adjusting to the new family arrangements (Cavanagh and Huston 2006; Mitchell et al. 2015). Prior research has found that these transitions might also influence child well-being through effects on the mental health of both parents (Cooper et al. 2009) and through changes in maternal parenting practices (Beck et al. 2010).^3^ Thus, changes in family structure, even if they involve the addition of a parental figure, may generate stress for the children as well, as they may create ambiguity in household rules, family relationships and parental expectations (Wu and Martinson 1993; Cavanagh and Huston 2006; Osborne and Mclanahan 2007).

The ambiguity in household rules may result in loser *social control* within the family and limit the opportunities for *social learning*, which may be especially important for children’s physical health in early childhood. Social control, in this context, refers “to direct and purposeful attempts to control and monitor, regulate another’s health behaviour”, and social learning is more “indirect internalisation of norms and meanings of a social role that influence health behaviours” (Reczek et al. 2014). Early theoretical work suggested that strong family ties and parenthood are important for children’s health outcomes due to social control processes (Umberson 1987); however, empirical evidence has been contradictory, and depended on child’s gender, as girls might be more influenced by social control processes carried by mothers regarding eating behaviours and exercise (for a review of the literature, see Reczek et al. 2014). While empirical studies on social control processes have not considered these specific family transitions explicitly, it is plausible that fathers’ entry will alter mother’s social control on their children and the environment for social learning, which is important for children’s health behaviour.

### Social Versus Biological Father?

Although having a father in the household may have positive effects on a range of child outcomes, no study so far has distinguished between the diverse effects of the biological father’s *entrances* and non-biological or social fathers’ *entrances* to the family environment of the child (Mitchell et al. 2015). From the point of view of economic resources, all else being equal, social and biological fathers’ entrance should improve equally the resources available to children relative to those growing up continuously with a lone mother. There is limited theoretical and empirical work on the question of whether subsequent intra-household *material* resource allocation operates differently between stepfamilies and biological families (Ginther and Pollak 2004). Family sociologists argued that biological fathers will invest more economic resources in children compared to social fathers because biological fathers are both socially and legally obliged to do so, while economic obligations of social fathers are not fully institutionalised (Furstenberg and Cherlin 1991; Cherlin and Furstenberg 1994).

There is a growing literature on the differences between biological and stepfamilies in terms of parenting practices and involvement (e.g. Berger et al. 2008; Bzostek 2008; Hofferth and Anderson 2003). In theory, a step-parent may help with childcare responsibilities and improve time/parenting resources, although evidence suggests that the supervision of children does not increase with a stepfamily formation (Thomson et al. 2001). Evolutionary perspectives suggest that biological resident fathers will invest more in children compared to resident social fathers because the former group invests in children as a form of “relationship and bonding effort”, while the latter group invests in children as a form of “mating effort” concerning their relationship with childs’ mother (see the literature cited in Berger et al. 2008, p. 3; Sweeney 2010). Studies from the USA have indeed found that stepfathers are generally less involved than biological fathers, but there is considerable variation according to marital status, family composition and father’s marital history (e.g. Hofferth and Anderson 2003; Berger et al. 2008).

Regarding the instability-stress perspective, in the case of stepfamilies, we expect to find that the need for adjustment and stress levels might be greater because the role of the father may be more difficult to establish (Coleman et al. 2000). This could be particularly the case if stepsiblings are involved (Hetherington and Kelly 2002). More consistent evidence suggests that educational outcomes of children are affected by the presence of stepsiblings and half siblings regardless of the biological status of parents (Ginther and Pollak 2004; Halpern-Meekin and Tach 2008).

The final family trajectory we consider includes unstable biological families, where a father enters the family but leaves again before the child turns 7. We expect that the exposure to a biological family formation in early childhood is important and may constitute a boost to their well-being in general, but this group experiences a family dissolution, which is consistently found to affect negatively *all* child outcomes as outlined at the beginning of this section. Thus, it is difficult to make predictions about which of these effects dominates in practice for children in this family trajectory.

### Summary of Predictions

Even if it is hard to predict the sign of the associations between each family trajectory and the different outcomes due to many offsetting mechanisms discussed above, we can broadly summarise our expectations by family trajectories as follows: first, we expect that children born to a lone mother who experience a stable biological father’s entry are likely to fare better in all the outcomes considered in this study than those that live continuously with a lone mother. The resources in the family increase, which may be positively associated with cognitive outcomes. There may be improvements in social learning and control environment and clearer roles, which may be positively associated with health outcomes. Biological fathers are likely to invest in their children, and subsequent stability may correlate with improved socio-emotional behaviour.

Second, children that experience a stepfather’s entry are likely to be similar to children growing up with lone mothers, especially regarding cognitive and socio-emotional outcomes, given that supervision and father’s involvement might be limited. The added instability experienced and potential difficulty in role adjustment, which may disrupt social control and learning environment, could altogether offset the benefits of increase in family resources.

Third, children in family trajectories characterised by the formation and then dissolution of the biological parents’ union may fare worse, especially in cognitive and socio-emotional outcomes, than children growing up in stable lone-mother family. In fact, the instability generated by the father’s entry and subsequent exit from the household, changes in family resources due to parental separation, and disruption in social learning environment may be negatively associated with children’s cognitive and socio-emotional outcomes. This negative association would potentially offset the positive association between biological father’s temporary entry into the child’s home and child outcomes.

## Data

The paper uses data from the Millennium Cohort Study (MCS), a UK longitudinal cohort study of around 19,000 children who were born in the UK between September 2000 and January 2002. The sample was selected from a random sample of electoral wards with a stratified sampling strategy to ensure a sufficient number of observations from all four UK countries and from disadvantaged and ethnically diverse areas (Hansen 2012). For this reason, the analyses used sample weights to adjust for the unequal probability of being sampled and the stratified and clustered sample design. The first sweep of data was collected when the cohort members were around nine months old, and subsequent sweeps of data were collected when the children were around three, five, seven and 11 years old. During home visits, interviewers collected information about a range of factors, including demographic characteristics (the relationship status was recorded at each sweep of data collection), socio-economic circumstances, different measures of child well-being and the parent’s behaviours.

### Sample

Our sample is made up of two groups: children whose mother was neither married nor cohabiting with a partner at the time of birth and all children who were born into a household where their biological parents lived together and remained together for the first seven years of life of the child. The first group is our population of interest; the second group is considered to provide context.

To identify children of lone mothers, we consider information from the first sweep of the MCS. In the first sweep of the MCS, the main respondent was asked: “What was your relationship with (the child’s name)’s father at the time (he/she) was born?”. Mothers could answer one of the following: married and living together, cohabiting/living as married, separated, divorced, closely involved, just friends, not in any relationship. We select the group of mothers who answered one of the following: closely involved, just friends, not in any relationship. We call this group “lone mothers at birth”. It is worth noting that these women might have been romantically involved with the father of the child or with someone else when the child was born; however, they were neither married nor cohabiting with a partner. Thus, the definition of lone mother is based on residency with a father figure. We then follow the children of these mothers until sweep 4, that is, when they were around seven years old. Partnership status at subsequent waves is constructed through information provided in the household grid and on a survey question about the relationship between the main and (if present) partner respondent. We retain only those observations for which we have a valid interview at every sweep of data collection.

The total number of children born to a lone mother in the MCS is 3285. Using population weights, we estimate that in the UK population in 2000, about 14.7% of children were born to lone mothers, according to our definition, which is close to the figure from official statistics. We lose 1718 children because of attrition and are left with 1567 children. From this figure, we had to exclude 278 cases because of rare or unclassifiable trajectories, leading to a sample size of 1289. Lastly, we had to drop 120 observations because of missing outcomes. The final number of children in this group is 1169. This figure corresponds to 11.4% of all the MCS children.

One striking finding is the high rate of attrition in this subset of the population. More than half of children born to a lone mother who participated in the MCS at sweep 1 had dropped out by sweep 4. A more careful analysis showed that the largest rate of attrition occurs between Sweeps 1 and 2 when 29.3% of the observations are lost. Between Sweeps 2 and 3 and between 3 and 4, the attrition rate remains constant at about 17%. This should be compared with the overall attrition rate for MCS that is 39.1%. This finding confirms that we are dealing with a hard to reach population. To account for the attrition, we used non-response weights although we cannot exclude the possibility that the results may have been subject to bias.

To provide context and as a comparison, we include the group of children whose biological parents were together at the time of birth of the child and did not separate at any point before the collection of Sweep 4. After considering attrition, and availability of outcome variables, we end up with a subsample of 6161 children who fall into this group. Thus, the total analytical sample consists of 7330 children.

Finally, it is important to note that the US literature on family resources across family types makes a clear distinction between cohabitation and marriage. Nonetheless, in this study we group cohabitation and marriage together. We have done this in order to maximise our sample size and because in the UK unmarried cohabitations have been consistently found to be more stable and marriage-like than cohabitations in the USA (Kiernan et al. 2011).

### Family Trajectories

From the sample of children born to lone mothers, we construct mothers’ union trajectories in the first seven years of life of the child (Table 1). We base the construction of the trajectories on two criteria. First, the trajectories need to be theoretically relevant. We are interested in examining the mothers’ partnership experiences, and in doing so we distinguish biological fathers from stepfathers and between stable and unstable unions. Second, because the trajectories need to provide an adequate sample size, we have to exclude rare and unclassifiable trajectories.^4^

**Table 1:** Family trajectories from birth until age 7 for children born to lone mothers. *Source* Millennium Cohort Study

Family trajectory					Trajectory name	Sample size	Trajectory size (%)	% of total UK births in 2000
Birth	9 months	3 years	5 years	7 years				
Continuously lone mothers					L		534 (46%)	5.4
Lone	Lone	Lone	Lone	Lone		534		
Lone to stable biological families					L-B		370 (32%)	3.4
Lone	Bio	Bio	Bio	Bio		183		
Lone	Lone	Bio	Bio	Bio		139		
Lone	Lone	Lone	Bio	Bio		47		
Lone	Lone	Lone	Lone	Bio		1		
Lone to stable stepfamilies					L-S		113 (10%)	1.2
Lone	Step	Step	Step	Step		8		
Lone	Lone	Step	Step	Step		47		
Lone	Lone	Lone	Step	Step		56		
Lone	Lone	Lone	Lone	Step		2		
Unstable biological families					L-B-L		152 (13%)	1.5
Lone	Bio	Bio	Bio	Lone		12		
Lone	Lone	Bio	Bio	Lone		17		
Lone	Lone	Lone	Bio	Lone		8		
Lone	Bio	Lone	Lone	Lone		65		
Lone	Bio	Bio	Lone	Lone		24		
Lone	Lone	Bio	Lone	Lone		26		
Total							1169 (100%)	11.4^a^
Children not included in any of the above trajectories							252	

Among the remaining children not included in any category (252) the most frequent cases are the following. For 115 children we are unable to classify the relationship status of the mother. The second most frequent trajectory not included in the sample is that of a mother who was single when her child was age seven, but who had formed at least one union with a stepfather in the seven years since the birth of the child. This trajectory comprises 50 observations

^a^The percentage of children born to a lone mother in the MCS is 14.7%; however, only a subsample of them enter our analytical sample, due to attrition and missing items

The trajectories included in the analysis are the following: children who live with a lone mother for the first seven years of their life, which we refer to as trajectory L; children who are born to lone mothers but then live with their biological father and do not experience the dissolution of the parents’ relationship at any time until they are 7, which we refer to as trajectory L-B (from the pattern Lone-Bio); children who are born to lone mothers and then transition to a stepfamily and later experience the dissolution of the partnership, which we refer to as trajectory L-S (from Lone-Step); at last, children who are born to a lone mother, then live with their biological father and experience the dissolution of the partnership, so that the mother is again single when they are 7 years old; we refer to this group with the acronym L-B-L (from the sequence Lone-Bio-Lone). The largest trajectory is L; 5.2% of all the MCS children live continuously with a lone mother from birth until age 7. Although the main focus of the paper is the comparison of outcomes among children born to a lone mother (L, L-B, L-S, L-B-L), we also compare each of these trajectories with a more traditional household type in which the child lives continuously with both biological parents until age seven hereafter referred to as B (Bio).

### Analytical Strategy

The analytical strategy is divided into two sections. The first section aims to investigate the heterogeneity of family backgrounds among children who were born to lone mothers. The goal of the second section is to investigate the association between a range of child outcomes and the four trajectories. Children who were living with a mother who formed a union with a father (trajectories L-B, L-S and L-B-L) are compared to children who were living continuously with a lone mother until age seven (the reference trajectory L). To provide context, in both sections, we also compare each of the trajectories with a more traditional household type in which the child lives continuously with both biological parents until age seven (trajectory B).

The formation or dissolution of a union is related to a number of factors, including maternal and household attributes that potentially are also relevant for child well-being. In the multivariate models, we include a set of background characteristics of the mother that account for some of these attributes. Thus, we are interested in identifying the association between the family trajectories and child outcomes after the confounding effect of background characteristics is taken into account. It is important to note that our results represent descriptions of the associations rather than the causal effects of those transitions; even in this already selected group of children there may be further selection processes associated with the mothers’ characteristics and with the well-being of their children that may make them more likely to experience one type of transition than another. Because our interest lies in accounting for factors that determine selection into different trajectories, we want to control for background variables that are measured as early as possible. Thus, we include variables that are measured at birth (and asked retrospectively at Sweep 1)—whenever possible—or at the first sweep, when the children were nine months old. In section 4.2, we provide detailed descriptions of the variables included and the time at which they are measured. To allow comparison of effect sizes across outcomes, we compute partial correlation coefficients. We computed them in a three-step process. First, we regressed each outcome on all explanatory variables apart from the variable of interest and took residuals. Second, we regressed the variable of interest on all explanatory variables and took the residuals. Third, we regressed the residuals from step 1 on the residuals from step 2. The R squared of this last regression corresponds to the partial correlation coefficient. We apply survey weights to all the regressions.

## Measures

### Outcome Measures

We select six outcomes to capture three dimensions of child well-being: health, cognitive development and socio-emotional well-being. All outcomes were measured when the children were seven years old. These outcomes have been widely used as markers of child well-being, and they are associated with well-being later in life (e.g. Feinstein 2003; Goodman 2001; Guo and Chumlea 1999). The description of the measurement and definition of all outcomes is presented in the  Appendix Table 8.

To measure the health of the child, we consider whether the child was obese. This is a binary indicator, and it is derived from weight and height measurements (Cole et al. 2000). Obesity is defined using the International Obesity Taskforce (IOTF) body mass index (kg/m^2^) cut points, which are age and gender specific. At each sweep of the MCS, children were weighed without shoes or outdoor clothing using Tanita HD-305 scales (Tanita UK Ltd, Middlesex, UK), and the weights were recorded in kilograms to one decimal place. Heights were obtained using the Leicester Height Measure Stadiometer (Seca Ltd, Birmingham, UK) and were recorded to the nearest millimetre.

To measure cognitive well-being, we consider the results of two standardised tests taken from the British Ability Scale (Hill 2005), namely pattern construction and word reading, and one test from the National Foundation for Education Research (NFER), which assesses children’s mathematical skills. All three items are measured on a continuous scale.

To measure socio-emotional well-being, we use two scales obtained from the Strengths and Difficulties Questionnaire (Goodman and Goodman 2009), which was completed by the main respondent at the time of the interview. We consider two scales that measure how prone children are to experiencing internalising (e.g. anxiety, depression) and externalising (e.g. attention deficit, uncooperative behaviour) disorders, which we analyse separately, given that their associations with our trajectories show different patterns (see Table 2). We recoded both outcomes so that higher values indicate a lower risk of suffering from internalising/externalising problems; both outcomes are measured on a continuous scale.

**Table 2:** Mean outcomes measured at age 7, broken down by family trajectory. *Source* Millennium Cohort Study

	Health		Cognitive		Socio-emotional	
	Obesity	Pattern construction score	Word reading score	Number skills score	Internalising scale	Externalising scale
Range	0–1	20–80	55–145	69–136	−17 to 0	−20 to 0
L	0.1 (0.02)	49 (0.55)	107.75 (1.06)	93.55 (1.01)	−3.81 (0.17)	−6.04 (0.20)
L-B	0.062 (0.01)	51.4 (0.78)	108.29 (1.26)	96.66 (1.18)	−3.13 (0.18)	−5.98 (0.24)
L-S	0.08 (0.04)	49.39 (1.37)	102.18 (2.31)	93.87 (1.70)	−4.06 (0.41)	−5.89 (0.42)
L-B-L	0.04 (0.02)	49.84 (0.96)	104.51 (1.54)	94.83 (1.70)	−3.99 (0.29)	−6.23 (0.37)
B	0.05 (0.00)	54.66 (0.23)	115.37 (0.34)	100.32 (0.37)	−2.32 (0.05)	−4.06 (0.06)

Standard errors in parentheses. Estimates obtained using Sweep 4 survey weights

In Table 2, we report sample mean estimates of the outcome variables, broken down by trajectory. For all outcomes, children in trajectory B are better off than children of lone mothers. However, there is variation within the trajectories of children born to a lone mother. About 10% of children in trajectory L are obese, but only 6% in trajectory L-B and 4% in trajectory L-B-L. For the cognitive scores, children in trajectory L-B have higher average scores than all other trajectories for children born to a lone mother. For socio-emotional outcomes, we find different patterns for the internalising and externalising scales. For instance, although children in trajectory L-S have the worse scores in the internalising scale, they have the best scores in the externalising scale.

## Background Characteristics of Family Trajectories

The next analytical step consists in exploring the relationship between family background and the different partnership trajectories. First, we look at the biological parents’ relationship at the birth of the child and investigate whether the nature of this relationship is related to the mothers’ subsequent marital history. Second, we compare households that belong to each of the trajectories under study with each other and with the households in trajectory B in terms of a set of background covariates.

### Are Children Born to Lone Mothers All the Same?

The MCS provides information on the relationship status of the biological parents of the child when the child was born. In particular, we know whether the parents were romantically involved but not married or cohabiting, were friends or were not in a relationship. We believe that at least a subset of couples who were romantically involved but not cohabiting may have been in a living apart together (LAT) relationship. In the literature, this type of relationship is defined as “unions between unmarried partners who live in separate households but identify themselves as part of a couple” (Strohm et al. 2009). However, without having further information, we are not able to characterise more precisely the nature of these non-residential relationships.

**Fig. 1:**
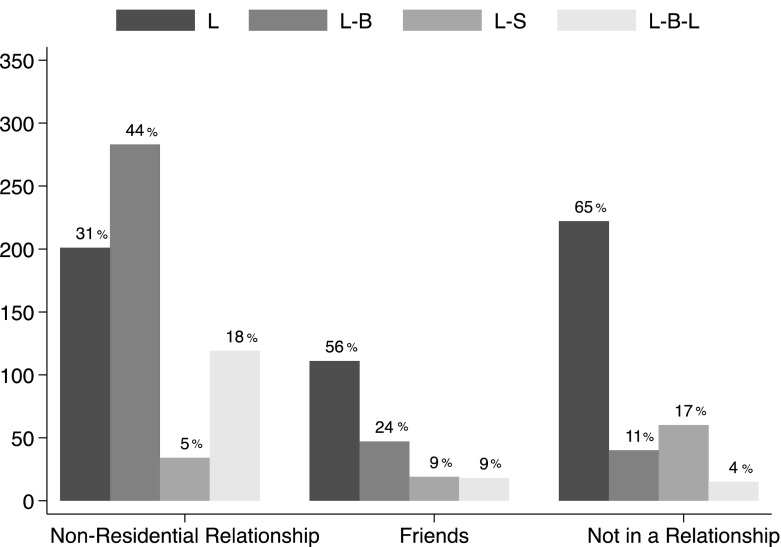
Distribution of trajectories conditional on the relationship between biological parents at birth (headcounts and percentages). The height of the *bar* and the *vertical axis* identify the count of cases. The labels on top of *each bar* give the percentage of respondents in each trajectory conditional on each relationship at birth type. For instance, the *first bar* on the *left* shows that about 200 mothers were in trajectory L (continuously lone mothers) and in a non-residential relationship with the biological father of the child when the child was born; 31% of all mothers who were in a non-residential relationship at the time of birth were in trajectory L (continuously lone mothers). *Source* Millennium Cohort Study

In Fig. 1, we report the number of respondents who ended up in each of the trajectories, broken down by relationship status between the biological parents at birth of the child. For the mother, being in a non-residential relationship with the biological father was associated with a higher probability that she would marry or cohabit with the father. This suggests that in these families (L-B and L-B-L) it is likely that the father was involved in the life of the child since birth. Mothers who were not in a relationship with the biological father were the most likely to start a stepfamily. Mothers who remain continuously single (L) were equally likely to have been in a non-residential relationship or not in a relationship at the birth of the child.

In Table 3, we describe the trajectories in terms of the mother’s/family’s characteristics measured at nine months or, when available, at birth. The background variables and how they are measured are described in detail in “Appendix” Table 7.^5^

**Table 3:**
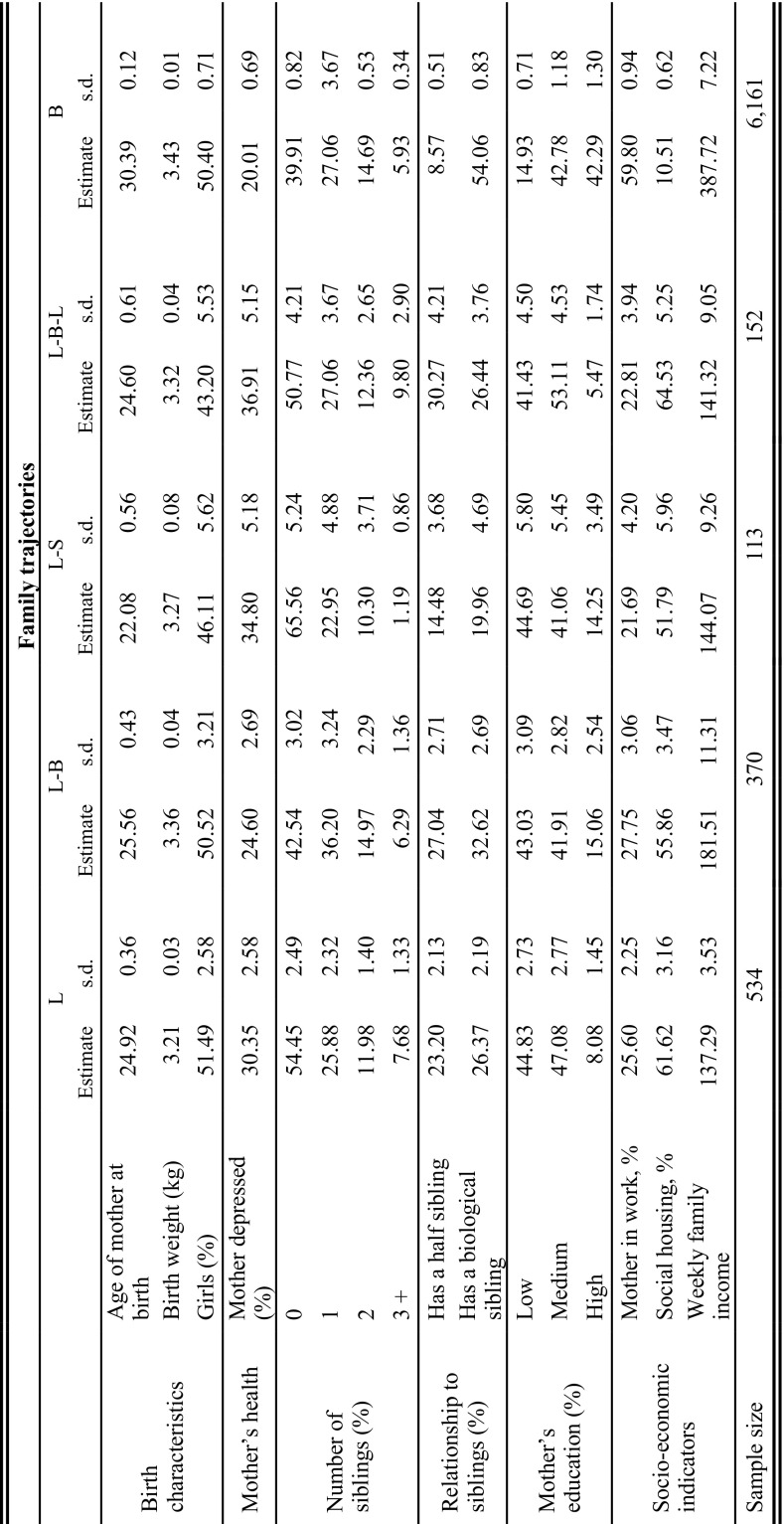
Descriptive statistics of background variables measured at 9 months or at birth broken down by family trajectory, means and percentages

Birth weight is a marker of health status in childhood and is associated with outcomes later in life (Druet et al. 2012). The age of the mother at birth is associated with the child outcomes (e.g. Goisis 2015) and with her future partnership formation patterns and the stability of those partnerships (e.g. Rindfuss and St. John 1983). Similarly, the negative relationship between maternal post-natal depression and children’s well-being is well known (e.g. Luoma et al. 2001), while mental health disorders have been shown to be associated with both a lower probability of forming a partnership and a higher probability of marital disruption (e.g. Whisman et al. 2007). Mother’s education, labour force participation, weekly household income and social housing residency are all socio-economic indicators that are known to be associated with child well-being (e.g. Cooper and Stewart 2013) and with family structure (e.g. Lichter et al. 2003).

The figures in Table 3 show that the households of children born to lone mothers differed from those of children whose parents were continuously married/cohabiting between birth and age seven (trajectory B). Reassuringly, the results reveal associations that are well known in the literature (e.g. McLanahan and Sandefur 1994, Osborne et al. 2012). Lone mothers at birth belonged to a particularly disadvantaged socio-economic group: when the child was nine months old, the average household income of these families was lower than that of other types of families and mothers were on average quite young and poorly educated. The children born to lone mothers were much more likely to live in social housing. Another important difference was the labour force participation rate of mothers, as the lone mothers at birth were less likely to have had a job during pregnancy.

Although there is considerable variation among all of the children born to lone mothers depending on their specific family trajectory, it is difficult to identify a gradient. In terms of socio-economic and health measures, mothers of children in trajectory L-B appeared to be the group with the best outcomes. These households have higher weekly family income, and mothers are more likely to be highly educated, less likely to suffer from depression and have the highest labour force participation rate among lone mothers. However, we also found a relatively high rate of social housing residency. In trajectory L-B-L, we found the highest prevalence of maternal depression, social housing residency and low average weekly family income and employment participation; however, the majority of these mothers are at least medium educated. Although families in trajectories L-S had low average weekly household income, they had the lowest prevalence of social housing residency and a relatively large proportion of mothers in this trajectory had a high level of education (14%). Finally, trajectory L seems to include the worst off households, as measured by the very high prevalence of social housing residency and maternal depression, the lowest weekly family income, low labour market participation and a very high proportion of mothers with low education.

The heterogeneity of the family backgrounds of children at birth (or at nine months old) may be associated with both their outcomes at age seven and the relationship trajectory of the mother. Thus, in the next section, we present partial associations between trajectories and child outcomes after controlling for the confounding effects of background variables.

## Associations Between Family Trajectories and Child Outcomes

Table 4 shows the unconditional and conditional associations between the child outcomes and the family trajectories. The reference trajectory is children living continuously with a lone mother (L). We show the models comparing the subgroups of children born to lone mothers and children living with continuously married parents (trajectory B). Separate analyses of the subsamples of children born to lone mothers show qualitatively similar results (available upon request). In Table 4, Models (a) show the association between each outcome and the trajectories and only include a control for the child’s sex and Models (b) include the full set of controls. Nevertheless, most of the significant associations revealed in the bivariate analyses are robust to the inclusion of background variables measured at baseline.

**Table 4:** Association between family trajectories of children born to lone mothers and children growing up in intact families and outcomes at 7 years old. *Source* Millennium Cohort Study

	Obesity^a^		Pattern construction score^b^		Word recognition score^b^		Number skills score^b^		SDQ internalising scale^b^		SDQ externalising scale^b^	
	(1a)	(1b)	(2a)	(2b)	(3a)	(3b)	(4a)	(4b)	(5a)	(5b)	(6a)	(6b)
	*β*/(SE)	*β*/(SE)	*β*/(SE)	*β*/(SE)	*β*/(SE)	*β*/(SE)	*β*/(SE)	*β*/(SE)	*β*/(SE)	*β*/(SE)	*β*/(SE)	*β*/(SE)
L-B (*reference L*)	−0.52* (0.30)	−0.50 (0.32)	2.42*** (0.92)	1.56* (0.92)	0.57 (1.56)	−0.58 (1.45)	3.10** (1.34)	1.85 (1.25)	0.67*** (0.25)	0.48* (0.25)	0.06 (0.31)	−0.15 (0.30)
L-S	−0.25 (0.59)	−0.17 (0.58)	0.41 (1.48)	0.09 (1.40)	−5.41** (2.27)	−5.76*** (2.04)	0.26 (1.86)	−0.27 (1.74)	−0.26 (0.43)	−0.22 (0.41)	0.20 (0.44)	0.24 (0.41)
L-B-L	−0.95** (0.47)	−0.99** (0.47)	0.89 (1.12)	0.86 (1.13)	−2.99* (1.61)	−2.74* (1.58)	1.18 (1.59)	1.14 (1.63)	−0.19 (0.34)	−0.19 (0.34)	−0.09 (0.44)	−0.05 (0.42)
B	−0.81*** (0.22)	−0.42 (0.27)	5.66*** (0.58)	1.01* (0.60)	7.65*** (1.05)	−0.11 (1.07)	6.75*** (1.01)	−0.38 (1.04)	1.48*** (0.17)	0.33* (0.19)	1.99*** (0.20)	0.59*** (0.22)
Age of mother at birth		0.02* (0.01)		0.06* (0.03)		0.22*** (0.05)		0.01 (0.05)		0.01* (0.01)		0.04*** (0.01)
Birth weight		0.32*** (0.11)		1.85*** (0.24)		1.63*** (0.42)		2.19*** (0.33)		0.17*** (0.07)		0.31*** (0.08)
Girl	0.25** (0.11)	0.27** (0.12)	0.59** (0.28)	0.95*** (0.27)	2.91*** (0.46)	3.38*** (0.44)	−1.26*** (0.43)	−0.76* (0.41)	0.01 (0.06)	0.06 (0.06)	1.14*** (0.09)	1.21*** (0.08)
Mother depressed		0.23 (0.14)		−0.26 (0.34)		−1.25** (0.53)		−0.82 (0.51)		−0.55*** (0.09)		−0.46*** (0.11)
No. of siblings		−0.05 (0.11)		0.03 (0.28)		−1.68*** (0.45)		−0.33 (0.45)		−0.04 (0.08)		0.25** (0.10)
Biological siblings		0.16 (0.20)		−0.32 (0.45)		−0.15 (0.71)		0.69 (0.67)		0.52*** (0.12)		−0.17 (0.17)
Half siblings		−0.22 (0.26)		−1.17** (0.56)		−1.33 (0.99)		−1.52* (0.87)		0.13 (0.17)		−0.79*** (0.20)
Mother’s education: medium (ref. low education)		−0.28* (0.17)		1.53*** (0.46)		2.88*** (0.69)		2.48*** (0.66)		0.51*** (0.12)		0.55*** (0.13)
Mother’s education: high (ref. low education)		−0.82*** (0.22)		3.28*** (0.48)		6.30*** (0.73)		5.56*** (0.71)		0.56*** (0.12)		0.92*** (0.15)
Mother working		0.09 (0.16)		−0.35 (0.38)		−0.14 (0.56)		0.83 (0.53)		0.34*** (0.09)		−0.20* (0.12)
Social housing		0.56*** (0.19)		−1.71*** (0.50)		−1.59** (0.78)		−1.08 (0.77)		−0.47*** (0.13)		−0.46*** (0.15)
Log income		−0.13 (0.13)		2.01*** (0.33)		3.42*** (0.50)		3.62*** (0.46)		0.36*** (0.07)		0.37*** (0.10)
Constant	−2.33*** (0.22)	−3.60*** (0.78)	48.71*** (0.58)	32.07*** (2.69)	106.25*** (1.12)	80.1*** (3.28)	94.19*** (1.05)	68.54*** (3.10)	−3.81*** (0.17)	−6.61*** (0.48)	−6.62*** (0.20)	−10.44*** (0.58)
*n*	7330	7330	7330	7330	7330	7330	7330	7330	7330	7330	7330	7330

^a^Coefficients from logistic regressions expressed in log odds. ^b^ Figures are unstandardised coefficients from OLS regressions

Significance levels: *** 1%, ** 5%, * 10%

In Table 5, we provide effect sizes for all trajectories and for income, as a comparison, based on the results for the multivariate regressions in Table 4. The figures are partial correlation coefficients; they correspond to the proportion of variance of the outcome explained by each factor. They allow us to compare results across outcomes, which would not be possible from the unstandardised regression results in Table 4.

**Table 5:** Effect sizes from multivariate regressions in Table 4. *Source* Millennium Cohort Study

	Obesity	Pattern construction score	Word recognition score	Number skills score	SDQ internalising scale	SDQ externalising scale
L-B	0.08	0.07*	0.01	0.05	0.10*	0.01
L-S	0.01	0.01	0.19***	0.01	0.01	0.01
L-B-L	0.13**	0.01	0.05*	0.01	0.01	0.01
B	0.10	0.05*	0.01	0.01	0.09*	0.19***
Income^a^	0.01	0.81***	0.93***	1.31***	0.40***	0.29***

Figures are % of variance explained by each factor ^a^ logarithm of OECD equivalised weekly household income

Significance levels: *** 1%, ** 5%, * 10%

There is a strong negative association between having lived with a biological father (trajectories L-B and L-B-L) and obesity. Being in trajectory L-S is not negatively associated with obesity. This is consistent with the predictions of social control theory. The greater involvement of biological fathers than stepfathers can imply that enforcement of rules is easier in households with biological fathers, which has been shown to be beneficial for children’s physical health. The sizes of the effects are particularly large. From Table 5, we see that being in trajectory L-B-L explains 0.13% of the variation in obesity.

For numerical skills and pattern construction, we find a strong positive association with being in the L-B trajectory in the unadjusted models. However, the coefficient for numerical skills is no longer significant when controlling for baseline covariates. The results for the L-S and the L-B-L trajectories are not significant, although the sign of the coefficient is positive. By contrast, skills in recognising words are negatively associated with being in trajectories L-S and L-B-L. In other words, children in family trajectories involving multiple (L-B-L) or “complex” (L-S) transitions have worse reading skills than children who grow up in a more stable family environment (L or L-B). These results highlight that stress and family instability may be particularly detrimental for children’s cognitive outcomes. Although significant, the sizes of the effects for the cognitive outcomes are modest.

Children in the L-B trajectory are the only ones for whom we find a positive relationship for the internalising scale. This result is consistent with a stable increase in family resources associated with the entry of a biological father, especially in terms of emotional support to the mother and the child. Early and stable involvement of both parents in rearing the child may be associated with the reduction in the risk that the child will suffer from an internalising disorder, such as depression. In favour of this interpretation is the fact that we also find a positive association on the internalising scale for children in trajectory B compared to children in trajectory L.

For the externalising scale, we find that children in trajectory B have fewer behavioural problems (identified as an externalising disorder) than any of the children born to lone mothers, but no variation among any of the trajectories of children born to lone mothers. In other words, the positive association between behavioural problems and living with a lone mother holds, regardless of whether a father figure subsequently enters or exits the household. This finding is consistent with the previous literature, which showed that, on average, children of single mothers have more behavioural difficulties than children who live with two parents (e.g. Jones et al. 2013; Mitchell et al. 2015). It is unclear which (set of) mechanisms may explain this negative finding. The relative higher stability and resources in families L-B that contribute to explain health and cognitive outcomes do not seem to be a valid mechanism for behavioural problems (Table 6).

**Table 6:** Summary of findings

	Better than L				Worse than L				Not significant			
	L-B	L-S	L-B-L	B	L-B	L-S	L-B-L	B	L-B	L-S	L-B-L	B
Health												
Obese			✓						✓	✓		✓
Cognitive												
Pattern construction	✓			✓						✓	✓	
Word recognition						✓	✓		✓			✓
Number skills									✓	✓	✓	✓
Socio-emotional												
Internalising scale	✓			✓						✓	✓	
Externalising scale				✓					✓	✓	✓	

Based on conditional models (columns b) in Table 4

### Sensitivity Analysis

In around 25% of the cases in trajectory L (132 children), the mother entered a partnership when the child was younger than nine months. From a theoretical point of view, it is not clear whether it is correct to categorise these children as born to a lone mother, because although the father was not residing with them at birth, the co-residence started soon after. When we move the subset of children who already lived with their biological fathers at 9 months from trajectory L-B to B, the results remain qualitatively unchanged (Appendix Table 9). In the case of the pattern construction score, we lose statistical significance, but the coefficients remain qualitatively unchanged, suggesting the lack of statistical significance is due to loss of power after reducing the sample sizes. This test suggests that this subset of children does not explain why children in trajectory L-B have better outcomes than other children born to lone mothers.

We consider the heterogeneity of trajectory L along two dimensions: the level of education of the mother and the relationship between the biological parents at the time of birth. When considering the mother’s education, we find no evidence that children of lone high-educated mothers have better outcomes than children of lone low- and medium-educated mothers (Appendix Table 10). Therefore, it does not appear that variation in socio-economic resources among L mothers is associated with variation in children’s outcomes. However, this could be explained by the small sample size of the group of lone highly educated mothers and loss of estimation precision; in fact, only 49 mothers in this trajectory are highly educated, while 233 have low level of education and 252 have medium level of education.

Some of the mothers in the L group had a non-residential relationship with the biological father, while others had no relationship at all with him. Therefore, the father’s degree of involvement and support could vary and potentially be associated with child outcomes. When considering the relationship with the biological father at the time of birth, we find weak evidence that outcomes of children in trajectory L depend on the nature of the relationship between the biological parents (Appendix Table 11). Children in trajectory L whose mother was in a non-residential relationship with the biological father have higher word recognition scores than children whose mother was not in a relationship with the biological father.

## Conclusions

The aim of this paper was to explore whether heterogeneity of family trajectories in early childhood matters for outcomes of children born to lone mothers. We found that heterogeneity does matter. First the results showed that, consistent with existing evidence, children who were born to lone mothers belonged to a lower socio-economic group than the children who were born and grew up in families with two biological parents. But more importantly, within the group of children born to lone mothers, there is variation in children’s well-being depending on the family trajectories they experienced between birth and age seven.

Compared to the children of continuously lone mothers, children whose biological father stably joined the household (L-B) fared better in terms of cognitive outcomes and socio-emotional outcomes. In fact, for all outcomes but the externalising scale, they did almost as well as children who have lived continuously in a two-biological parent household since birth (B). In contrast, children in trajectories characterised by living with a non-biological father (L-S) or who experienced the dissolution of a union (L-B-L) had outcomes similar to children of continuously lone mothers (L). These findings are in line with theories on the improvement in social control and parental resources, and relative stability as suggested in the literature.

However, the entry of a father figure to the household was not associated with improvement on all outcomes. For instance, there were only small differences in the socio-emotional well-being of the children who were living with a lone mother and the children who were living with a stepfather. Based on existing theories, this finding suggests that the benefits of improved resources and parenting input could be offset by the difficulties in adjusting to a new situation in the child’s home environment when a stepfather joins the family. This finding sheds light on the vulnerability of these children in addition to those associated with growing up continuously with a lone mother. These results also highlight the importance of stability of the home environment: the benefits of a father’s entry for children’s outcomes in different areas are clearest in our results if the father is biological and the union is stable.

Although these results suggest that different family trajectories matter for the variation in child outcomes, they only do so to a certain extent: for instance, although the results show that children whose biological father joins the household and forms a stable union with the mother (L-B) fare better, this applies for only two of the outcomes studied here (pattern construction and internalising scale). Similarly, children who grow up in a stable two-biological parent household (B) do better than children who live continuously with their lone mothers (L) only in three outcomes (pattern construction, internalising and externalising scales) and in four outcomes (pattern construction, word recognition, internalising and externalising scales) compared to children who experience more instability (L-B-L and L-S). Yet, we believe that finding just a few persistent associations between family trajectories and child outcomes highlights the importance of looking closely at the heterogeneity of children’s family experiences. Our analysis had an exploratory aim our findings suggest that future research should devote more attention to the trajectories that we have found to be most relevant for child outcomes and explore in more details the mechanisms behind these associations.

Our findings are partially in line with findings from the USA, even though many of these studies focus on children born to unmarried (cohabiting) mothers, rather than lone mothers, and their subsequent family trajectory. For example, several studies in the USA showed that children who were living with their mother and her cohabiting partner had outcomes that were similar to those of children who were growing up with a single mother only, and their outcomes were worse than those of children who were living with a stepfather (Sweeney 2010; Thomson and McLanahan 2012). The literature also shows that children who were born to unmarried mothers and experienced the dissolution of their parents’ relationship are more likely to have health problems (Heiland and Liu 2006). These US studies argue that each additional transition is associated with higher likelihood of behavioural, cognitive and health problems. Our findings are in line with this: children growing up in family trajectories characterised by instability (L-B-L) may have worse outcomes than children living continuously with a lone mother (L).

The findings of this paper are specific to the UK context, and they may not necessarily apply to other settings. The UK context is characterised by high rates of teenage pregnancies (ONS 2016), which are associated with lone motherhood at birth. A higher rate of lone motherhood in general is likely to make lone mothers less selected in the UK than in other European countries. This argument is supported by the fact that in international comparisons children of lone mothers in the UK fare better than in other countries (Wößmann 2015). The ethnic composition of the UK population is also different from that of other European countries. In the UK, for example, women of black Caribbean ethnicity are more likely than other groups to be lone mothers and experience multiple partnership transitions and instability (e.g. Kiernan et al. 2011). Nevertheless, sample size issues prevented us from exploring whether the results differ by ethnic groups; the experience and consequences of being a lone mother and the partnership trajectories experienced during the early childhood years might indeed vary across groups of the population.

Data quality also limits the external validity of the findings. The high level of attrition that characterises our sample of interest limits the extent to which we can confidently claim the findings apply to all lone mothers in the UK. Indeed, if more disadvantaged mothers and those with more complex family histories are more likely to leave the survey (Hansen 2012), then our findings may be a lower bound of the real association between family trajectories and child outcomes. In spite of this, the MCS is an appropriate data source to use to address our research question and no other survey in the UK allows for a sample of lone mothers at birth as large as the one collected in the MCS.

In this paper, we treated the different types of child outcomes as independent from each other; however, it may well be possible that, for example, poor performance in cognitive outcomes may be related to difficulties in socio-emotional well-being or health outcomes. Furthermore, health, cognitive and behavioural outcomes may be related to family trajectories with varying strengths at different ages. Thus, future research should analyse the relationship between these different outcomes measured longitudinally to have a complete picture of the role of family trajectories on child well-being in its different dimensions. This study, which focused on a broad set of outcomes, should be viewed as a first step in that direction.

The theoretical contribution of this study is to underscore the diversity of life experiences of children born to lone mothers and, as a consequence, the importance of exploring heterogeneity within this group. Categorisations are common practice in the social sciences, but the usefulness of dividing a population into categories rests upon the researchers’ ability to identify meaningful groups and the relevant sources of disadvantage they experience. In particular, this article stresses the importance of not considering children born to lone mothers as a unique stand-along category, but rather to document, analyse and consider changes (or lack thereof) in the mothers’ partnership trajectories after birth and the different sources of advantage/disadvantage that might be associated with them.

Finally, our findings are also relevant for policy-makers. Children living with a lone mother are a population targeted by social policies in virtually all Western countries. Our results show the merit in distinguishing between different groups of children born to lone mothers, according to the family trajectories they experience in early childhood, which might be associated with different types of disadvantage and need for interventions. This finding should be compared to most social policies, which instead tend to target the overall group of children living with lone mothers, irrespective of previous family history.

## Appendix

Tables 7 and 8.

**Table 7:** Description and measurement of background variables according to the time at which they are measured

Variable	Description	Time of measurement
Age of mother	The age of the mother at the birth of the child	At birth
Birth weight	The weight of the baby in kilograms, as reported by the mother	At birth
Girl	The sex of the child	At birth
Mother suffers from depression	Dummy variable that takes the value of 1 if the mother has ever been diagnosed with depression	At 9 months
Number of siblings	Number of siblings of the child (not including the child himself/herself). Both biological and stepsiblings are included	At 9 months
Has a half sibling	Dummy variable that takes the value of 1 if the child lives with a half sibling (a sibling with the same mother or the same father as the child)	At 9 months
Has a biological sibling	Dummy variable that takes the value of 1 if the child lives with a biological sibling (a sibling with the same biological mother and father as the child)	At 9 months
Mother’s education level	Variable that does not change over time. This variable is based on National Vocational Qualifications levels equivalised to the highest level of academic or vocational education attained. In the MCS it takes on six values: no education (respondent reports no qualifications), low education (O level/GCSE grades D–G or equivalent vocational qualification), medium–low education (GCSE grades D–G or equivalent vocational qualification), medium–high education (A levels or equivalent vocational qualification), high education (university degree) and overseas qualification. We recode it so that it takes on three values: low education (no education, low education, and overseas qualification), medium education (medium–low and medium–high education) and high education (university degree)	At 9 months
Mother in work	Dummy variable that takes the value of 1 if the mother had a paid job at any time during pregnancy (self-reported)	At birth
Social housing	Dummy variable that takes the value of 1 if the mother reports that the family are renting from the local authority or from the Housing Association	At 9 months
OECD equivalised weekly household income	Equivalised household income per week (using the OECD equivalence scale)	At 9 months

**Table 8:** Description and measurement of outcome variables

Health outcomes	
Obesity	Dummy variable that takes a value of 1 if the child is obese. Obesity is defined using the International Obesity Taskforce (IOTF) body mass index (kg/m^2^) cut points, which are age and gender specific. At each sweep of the MCS, children were weighed without shoes or outdoor clothing using Tanita HD-305 scales (Tanita UK Ltd, Middlesex, UK), and the weights were recorded in kilograms to one decimal place. Heights were obtained using the Leicester Height Measure Stadiometer (Seca Ltd, Birmingham, UK) and were recorded to the nearest millimetre
Educational outcomes	
Pattern construction score	Cognitive score that is part of the British Assessment Scale. The child constructs a design by putting together at squares or solid cubes with black and yellow patterns on each side. The child’s score is based on accuracy and speed. We use the *T*-scores, which are adjusted for the child’s age group and for the mean scores of the BAS norming group. This procedure leads to an interval level variable with a mean of 50 and a standard deviation of 10 within the norming sample of a given age group (Hansen 2012)
Word recognition score	Cognitive score that is part of the British Assessment Scale aimed at assessing children’s English reading ability. The child reads aloud a series of words presented on a card. The assessment consists of 90 words in total. We use standardised scores, adjusted for age group and norming the sample mean and the standard deviation (Hansen 2012)
Number skills score	Cognitive score that is based on the results of a test adapted from the National Foundation for Educational Research (NFER) Progress in Maths test. All of the children complete an initial test, and based on their performance they are redirected to questions with three levels of difficulty. The scores have been nationally age standardised to a mean of 100 and a standard deviation of 15 (Hansen 2012)
Socio-emotional outcomes	
SDQ internalising scale	Summary of the “emotion symptom scale” and “peer problems”. This variable has been recoded so that a one-unit increase corresponds to a decrease in the risk of developing internalising disorders (e.g. depression, loss of interest in activities)
SDQ externalising scale	Summary of “conduct problems” and “pro-social scale”. This variable has been recoded so that a one-unit increase corresponds to a decrease in the risk of developing externalising disorders (e.g. attention deficit hyperactivity disorder, conduct disorder)

Tables 9, 10 and 11.

**Table 9:** Association between family trajectories of children born to lone mothers and outcomes at 7 years old when children who were living with their biological father at 9 months are included in trajectory B instead of L-B. *Source* Millennium Cohort Study

	Obesity^a^	Pattern construction score^b^	Word recognition score^b^	Number skills score^b^	SDQ internalising scale^b^	SDQ externalising scale^b^
	*β*/(SE)	*β*/(SE)	*β*/(SE)	*β*/(SE)	*β*/(SE)	*β*/(SE)
L-B	−0.36 (0.29)	1.24 (0.81)	−0.50 (1.26)	2.09* (1.14)	0.36* (0.22)	−0.392 (0.25)
L-S	−0.12 (0.58)	0.09 (1.38)	−5.69*** (2.00)	−0.06 (1.69)	−0.23 (0.40)	0.15 (0.41)
L-B-L	−0.95** (0.47)	0.85 (1.11)	−2.66* (1.60)	1.35 (1.65)	−0.20 (0.33)	−0.15 (0.42)
B	−0.34 (0.25)	1.01* (0.53)	−0.01 (0.95)	−0.06 (0.94)	0.31* (0.17)	0.46** (0.21)
Age of mother at birth	0.02* (0.01)	0.06* (0.03)	0.22*** (0.05)	0.01 (0.05)	0.01* (0.01)	0.04*** (0.01)
Birth weight	0.32*** (0.11)	1.85*** (0.24)	1.63*** (0.42)	2.19*** (0.33)	0.17*** (0.07)	0.31*** (0.08)
Girl	0.27** (0.12)	0.96*** (0.27)	3.38*** (0.44)	−0.77* (0.41)	0.06 (0.06)	1.21*** (0.08)
Mother depressed	0.23 (0.14)	−0.26 (0.34)	−1.25** (0.53)	−0.81 (0.51)	−0.55*** (0.09)	−0.47*** (0.11)
No. of siblings	−0.05 (0.11)	0.03 (0.28)	−1.68*** (0.45)	−0.33 (0.45)	−0.04 (0.08)	0.26*** (0.10)
Biological siblings	0.15 (0.19)	−0.32 (0.45)	−0.15 (0.71)	0.67 (0.67)	0.52*** (0.12)	−0.16 (0.17)
Half siblings	−0.21 (0.26)	−1.16** (0.56)	−1.33 (1.00)	−1.49* (0.87)	0.13 (0.17)	−0.79*** (0.20)
Mother’s education: medium (ref. low education)	−0.28* (0.17)	1.54*** (0.46)	2.88*** (0.69)	2.47*** (0.66)	0.51*** (0.12)	0.55*** (0.13)
Mother’s education: high (ref. low education)	−0.82*** (0.22)	3.29*** (0.48)	6.30*** (0.74)	5.56*** (0.71)	0.56*** (0.12)	0.93*** (0.15)
Mother working	0.09 (0.16)	−0.35 (0.38)	−0.14 (0.56)	0.83 (0.53)	0.34*** (0.09)	0.21* (0.12)
Social housing	0.56*** (0.19)	−1.69*** (0.50)	−1.58** (0.78)	−1.03 (0.76)	−0.47*** (0.13)	−0.47*** (0.15)
Log income	−0.14 (0.13)	2.01*** (0.33)	3.40*** (0.49)	3.58*** (0.46)	0.36*** (0.07)	0.39*** (0.10)
Constant	−3.55*** (0.77)	32.09*** (1.97)	80.09*** (3.27)	68.54*** (3.10)	−6.60*** (0.48)	−10.43*** (0.58)
Observations	7330	7330	7330	7330	7330	7330

^a^Coefficients from logistic regressions expressed in log odds. ^b^ Figures are unstandardised coefficients from OLS regressions

Significance levels: *** 1%, ** 5%, * 10%

**Table 10:** Association between family trajectories of children born to lone mothers and outcomes at 7 years old separating highly educated lone mothers from low and middle educated. *Source* Millennium Cohort Study

	Obesity^a^	Pattern construction score^b^	Word recognition score^b^	Number skills score^b^	SDQ internalising scale^b^	SDQ externalising scale^b^
	*β*/(SE)	*β*/(SE)	*β*/(SE)	*β*/(SE)	*β*/(SE)	*β*/(SE)
L (high education)	0.56 (0.62)	0.37 (1.48)	1.63 (2.62)	0.91 (2.50)	0.13 (0.61)	0.22 (0.60)
L-B	−0.47 (0.33)	1.59* (0.94)	−0.44 (1.52)	1.92 (1.28)	0.49** (0.25)	−0.13 (0.31)
L-S	−0.14 (0.58)	0.13 (1.43)	−5.63*** (2.09)	−0.20 (1.78)	−0.20 (0.41)	0.26 (0.41)
L-B-L	−0.38 (0.29)	0.89 (1.14)	−2.60 (1.60)	1.21 (1.67)	−0.18 (0.33)	−0.03 (0.43)
B	−0.96** (0.47)	1.04 (0.65)	0.03 (1.15)	−0.30 (1.13)	0.34* (0.19)	0.61*** (0.23)
Age of mother at birth	0.02* (0.01)	0.06* (0.03)	0.22*** (0.05)	0.01 (0.05)	0.01* (0.01)	0.04*** (0.01)
Birth weight	0.33*** (0.11)	1.85*** (0.24)	1.63*** (0.42)	2.19*** (0.33)	0.17*** (0.07)	0.31*** (0.08)
Girl	0.27** (0.12)	0.95*** (0.27)	3.38*** (0.44)	−0.76* (0.41)	0.06 (0.06)	1.21*** (0.08)
Mother depressed	0.22 (0.14)	−0.26 (0.35)	−1.27** (0.54)	−0.82 (0.51)	−0.55*** (0.09)	−0.46*** (0.11)
No. of siblings	−0.05 (0.11)	0.03 (0.28)	−1.68*** (0.45)	−0.33 (0.45)	−0.04 (0.08)	0.25*** (0.10)
Biological siblings	0.16 (0.19)	−0.32 (0.45)	−0.14 (0.72)	0.69 (0.67)	0.52*** (0.12)	−0.16 (0.17)
Half siblings	−0.22 (0.26)	−1.17** (0.56)	−1.34 (1.00)	−1.51* (0.87)	0.13 (0.17)	−0.79*** (0.20)
Mother’s education: medium (ref. low education)	−0.29* (0.17)	1.53*** (0.46)	2.86*** (0.69)	2.47*** (0.66)	0.51*** (0.12)	0.55*** (0.13)
Mother’s education: high (ref. low education)	−0.85*** (0.21)	3.27*** (0.48)	6.25*** (0.73)	5.53*** (0.73)	0.55*** (0.12)	0.91*** (0.15)
Mother working	0.09 (0.16)	−0.35 (0.38)	−0.15 (0.56)	0.83 (0.53)	0.34*** (0.09)	0.20* (0.12)
Social housing	0.56*** (0.19)	−1.70*** (0.50)	−1.59** (0.78)	−1.08 (0.77)	−0.47*** (0.13)	−0.46*** (0.15)
Log income	−0.13 (0.13)	2.01*** (0.33)	3.43*** (0.50)	3.63*** (0.46)	0.36*** (0.07)	0.38*** (0.10)
Constant	−3.62*** (0.77)	32.03*** (1.96)	79.94*** (3.29)	68.46*** (3.12)	−6.62*** (0.48)	−10.46*** (0.58)
Observations	7330	7330	7330	7330	7330	7330

^a^Coefficients from logistic regressions expressed in log odds. ^b^ Figures are unstandardised coefficients from OLS regressions

Significance levels: *** 1%, ** 5%, * 10%

**Table 11:** Association between family trajectories of children born to lone mothers and outcomes at 7 years old breaking down lone mothers according to the relationship with the biological father at the time of birth of child. *Source* Millennium Cohort Study

	Obesity^a^	Pattern construction score^b^	Word recognition score^b^	Number skills score^b^	SDQ internalising scale^b^	SDQ externalising scale^b^
	*β*/(SE)	*β*/(SE)	*β*/(SE)	*β*/(SE)	*β*/(SE)	*β*/(SE)
L (friends)	−0.07 (0.52)	−0.53 (1.45)	2.34 (2.55)	−1.69 (2.20)	0.56 (0.43)	0.56 (0.54)
L (non-residential relationship)	0.34 (0.46)	1.41 (1.17)	5.72** (2.33)	0.86 (1.79)	0.47 (0.34)	0.21 (0.44)
L-B	−0.38 (0.47)	1.97* (1.07)	1.99 (1.92)	1.82 (1.49)	0.76** (0.33)	0.04 (0.40)
L-S	−0.05 (0.67)	0.51 (1.51)	−3.20 (2.28)	−0.30 (1.91)	0.07 (0.45)	0.44 (0.50)
L-B-L	−0.30 (0.44)	1.42* (0.82)	2.45 (1.54)	−0.41 (1.29)	0.61** (0.28)	0.79** (0.34)
B	−0.88 (0.61)	1.27 (1.34)	−0.17 (2.04)	1.10 (1.85)	0.10 (0.40)	0.14 (0.50)
Age of mother at birth	0.02* (0.01)	0.06* (0.03)	0.21*** (0.05)	0.01 (0.05)	0.01* (0.01)	0.04*** (0.01)
Birth weight	0.33*** (0.11)	1.86*** (0.24)	1.65*** (0.42)	2.20*** (0.33)	0.17*** (0.07)	0.30*** (0.08)
Girl	0.27** (0.12)	0.96*** (0.27)	3.41*** (0.44)	−0.76* (0.41)	0.06 (0.06)	1.21*** (0.08)
Mother depressed	0.22 (0.14)	−0.27 (0.35)	−1.27** (0.53)	−0.83 (0.51)	−0.55*** (0.09)	−0.46*** (0.11)
No. of siblings	−0.04 (0.11)	0.04 (0.28)	−1.68*** (0.44)	−0.32 (0.44)	−0.04 (0.07)	0.25*** (0.10)
Biological siblings	0.16 (0.19)	−0.33 (0.45)	−0.16 (0.71)	0.68 (0.67)	0.52*** (0.12)	−0.16 (0.17)
Half siblings	−0.22 (0.26)	−1.19** (0.56)	−1.37 (0.99)	−1.53* (0.87)	0.13 (0.17)	−0.79*** (0.20)
Mother’s education: medium (ref. low education)	−0.27* (0.17)	1.55*** (0.46)	2.95*** (0.69)	2.49*** (0.66)	0.52*** (0.12)	0.55*** (0.13)
Mother’s education: high (ref. low education)	−0.82*** (0.22)	3.29*** (0.48)	6.32*** (0.74)	5.56*** (0.71)	0.56*** (0.12)	0.92*** (0.15)
Mother working	0.09 (0.16)	−0.34 (0.38)	−0.11 (0.56)	0.85 (0.53)	0.34*** (0.09)	0.20* (0.12)
Social housing	0.56*** (0.20)	−1.69*** (0.50)	−1.61** (0.79)	−1.06 (0.77)	−0.48*** (0.13)	−0.46*** (0.15)
Log income	−0.13 (0.13)	2.01*** (0.33)	3.42*** (0.50)	3.63*** (0.46)	0.36*** (0.07)	0.37*** (0.10)
Constant	−3.70*** (0.83)	31.62*** (2.08)	77.50*** (3.48)	68.51*** (3.19)	−6.88*** (0.53)	−10.62*** (0.64)
Observations	7330	7330	7330	7330	7330	7330

Reference category is trajectory L (not in a relationship)

^a^Coefficients from logistic regressions expressed in log odds. ^b^ Figures are unstandardised coefficients from OLS regressions

Significance levels: *** 1%, ** 5%, * 10%

## Footnotes

1 For example, commonly used data sets such as NLSY79, NELS88, National Survey of Children [1976, 81, 87] covered childhood years spent during 1970s and 1980s.

2 More recent US data sets such as Fragile Families and NICHD Study of Early Child Care and Youth Development (SECCYD) are representative of children born in certain cities or locations.

3 One exception is to this literature that Osborne et al. (2012) argue that if the transition is experienced before the age of 1, the effect is positive on child outcomes.

4 Although sequence analysis is increasingly used to identify common family trajectories over the life course, it is not appropriate for our purpose for various reasons: first, we are not interested in clustering sequences that are *similar* to each other, using an algorithm based on distance, but in identifying children who experience *exactly the same* sequence. This is because of the theoretical reasons that we explained in the text. Second, in our data, we observe parental family transitions at four discrete data points. In the absence of variation in duration of events, the advantage of sequence analyses is reduced. Furthermore, our four trajectories alone already describe the family trajectories experienced by more than 80% of children born to lone mothers in the MCS.

5 We would have liked to explore whether the results vary by ethnic groups or to include ethnicity as a control variable. However, the small variations in ethnicity in the estimation sample (around 87% of the children in this sample are white) did not allow us to estimate the results separately or to include it as a covariate.
